# Increased circulating full-length betatrophin levels in drug-naïve metabolic syndrome

**DOI:** 10.18632/oncotarget.15102

**Published:** 2017-02-04

**Authors:** Dan Liu, Sheyu Li, He He, Chuan Yu, Xiaodan Li, Libo Liang, Yi Chen, Jianwei Li, Jianshu Li, Xin Sun, Haoming Tian, Zhenmei An

**Affiliations:** ^1^ Department of Endocrinology and Metabolism, West China Hospital, Sichuan University, Chengdu, Sichuan, China; ^2^ Department of Laboratory Medicine, West China Hospital, Sichuan University, Chengdu, Sichuan, China; ^3^ Chinese Evidence-Based Medicine Center, West China Hospital, Sichuan University, Chengdu, Sichuan, China; ^4^ Department of Gastroenterology, West China Hospital, Sichuan University, Chengdu, Sichuan, China; ^5^ Department of General Medicine, West China Hospital, Sichuan University, Chengdu, Sichuan, China; ^6^ Department of Gastrointestinal Surgery, West China Hospital, Sichuan University, Chengdu, Sichuan, China; ^7^ Department of Biomedical Polymer and Artificial Organs, College of Polymer Science and Engineering, Sichuan University, Chengdu, Sichuan, China

**Keywords:** betatrophin, lipasin, ANGPTL8, metabolic syndrome, Pathology Section

## Abstract

Betatrophin is a newly identified circulating adipokine playing a role in the regulation of glucose homeostasis and lipid metabolism. But its role in metabolic syndrome (MetS) remains unknown. Therefore, we aimed to compare the circulating betatrophin concentrations between patients with MetS and healthy controls. We recruited 47 patients with MetS and 47 age and sex matched healthy controls. Anthropometric and biochemical measurements were performed, and serum betatrophin levels were detected by ELISA. Full-length betatrophin levels in patients with MetS were significantly higher than those in controls (694.84 365.51 pg/ml versus 356.64 287.92 pg/ml; *P* <0.001). While no significant difference of total betatrophin levels was found between the two groups (1.20 0.79 ng/ml versus 1.31 1.08 ng/ml; *P* = 0.524). Full-length betatrophin level was positively correlated with fasting plasma glucose (FPG) (r = 0.357, *P* = 0.014) and 2-hour plasma glucose (2hPG) (r = 0.38, *P* <0.01). Binary logistic regression models indicated that subjects in the tertile of the highest full-length betatrophin level experienced higher odds of having MetS (OR, 8.6; 95% CI 2.8-26.8; *P* <0.001). Our study showed that full-length betatrophin concentrations were increased in drug-naïve MetS patients.

## INTRODUCTION

Metabolic syndrome (MetS) is a cluster of multiple metabolic disorders including abdominal obesity, hypertension, glucose intolerance and dyslipidemia [1]. The prevalence of MetS is increasing at alarming rates threatening people's health by placing them at a higher risk of cardiovascular diseases, stroke and kidney diseases. Although physical inactivity, overweight/obesity and insulin resistance are definite risk factors for MetS [2], the underlying causes remain inconclusive.

Betatrophin, also known as lipasin, angiopoietin-like protein 8 (ANGPTL8) and refeeding induced fat and liver (RIFL), is a newly identified circulating adipokine predominantly synthesized in the liver and adipose tissue [3–6]. Betatrophin was supposed to be a regulatory mediator of glucose homeostasis and lipid metabolism in previous studies [7, 8]. Yi and colleagues showed that betatrophin promotes beta cell proliferation and expansion in insulin resistant mice [9]. However, several studies failed to prove its anti-diabetic effect [10, 11]. Therefore, controversy remains concerning the role of betatrophin in glucose homeostasis and insulin sensitivity [12–14]. However, it is well accepted that betatrophin operates as a blood lipid regulator by inhibiting lipoprotein lipase activity either directly or indirectly through promoting ANGPTL3 cleavage [3, 5, 8, 10].

Since the discovery of betatrophin has raised new hope for therapeutic approaches or identification of a potential biomarker for metabolic disorders, there is an increasing interest in measurement of the circulating level of betatrophin in humans. However, their results are conflicting. Some studies reported increased circulating betatrophin levels in patients with type 2 diabetes mellitus (T2DM) [15–20] and obesity [15, 21, 22], whereas others found no difference [23, 24] or even a decrease [25] in these metabolic disorders. Our previous meta-analysis has shown that the association between circulating betatrophin level and T2DM was diverse in different body mass groups [26]. There are also many discrepancies in results regarding the association between betatrophin and various metabolic parameters related to metabolic diseases [15–25, 27, 28].

A recent prospective cohort study has demonstrated that decreased levels of circulating betatrophin were related to the development of MetS [29]. However, it remains unknown whether betatrophin metabolism is altered in patients with MetS, we aimed to evaluate the circulating betatrophin levels in MetS patients in the current study.

## RESULTS

### Characteristics of study participants

**Figure 1:**
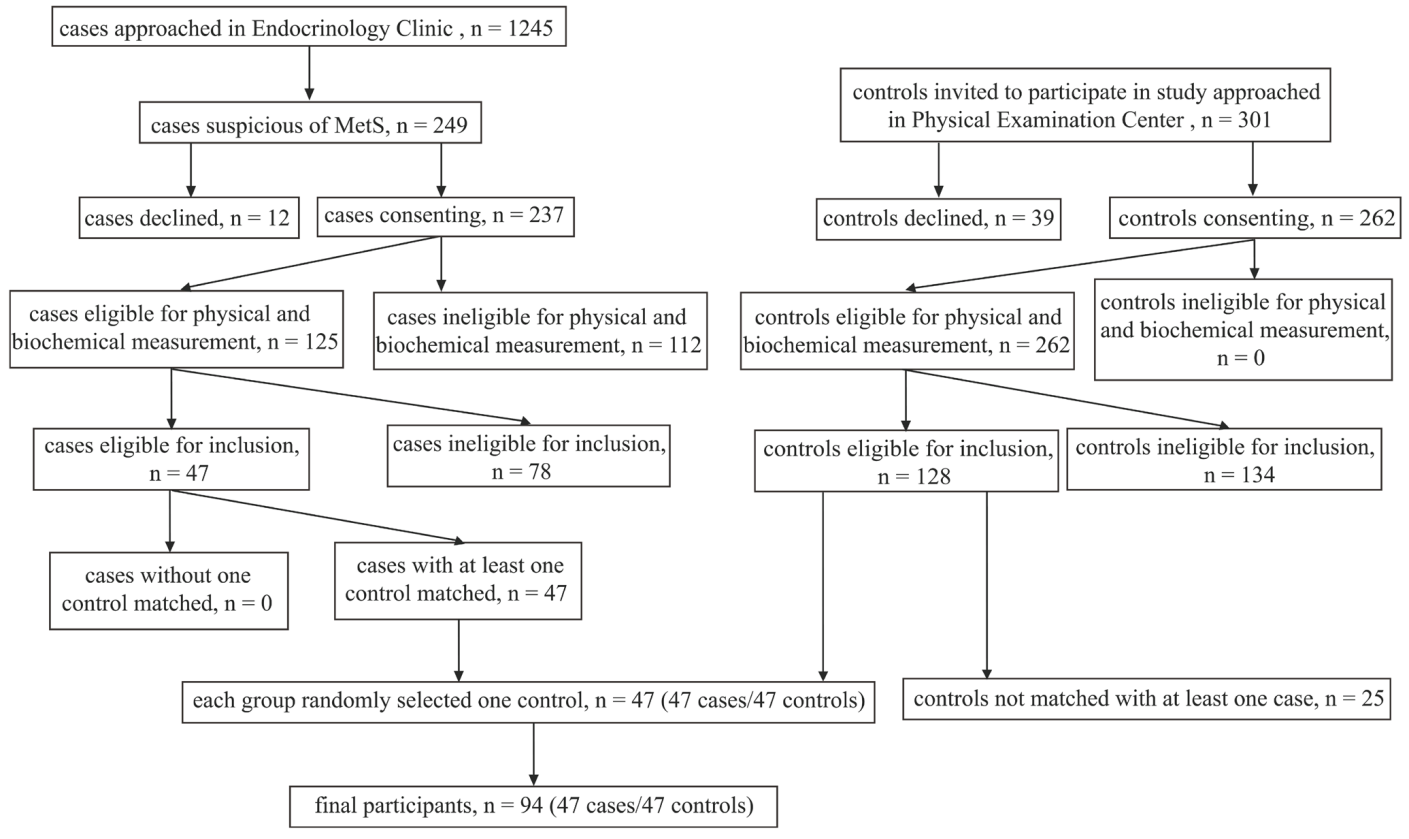
Flow chart of recruitment

As shown in Figure 1, we identified 47 MetS patients who met the inclusion criteria out of 1,245 cases approached in the Outpatient Clinic, and 47 age and sex matched healthy controls were recruited among 301 subjects approached in the Physical Examination Center during the enrollment period. The baseline characteristics of the two groups (MetS and healthy control) were summarized in Table 1. Since participants were matched for age and gender distribution, both were similar between the two groups. There was no statistically significant difference in low-density lipoprotein cholesterol (LDL-C), creatinine, estimated glomerular filtration rate (eGFR), alkaline phosphatase (ALP), total bilirubin (TBIL), direct bilirubin (DBIL), indirect bilirubin (IBIL) between groups. Body mass index (BMI), waist-to-hip ratio (WHR), fasting plasma glucose (FPG), hemoglobin A1c (HbA1c), triglyceride (TG), total cholesterol (TC), uric acid (UA), alanine transaminase (ALT), aspartate transaminase (AST) and γ-glutamyl transpeptidase (GGT) in MetS group were significantly higher than those in control group (*P* < 0.01, respectively), while high-density lipoprotein cholesterol (HDL-C) and albumin (ALB) were significantly lower (*P* < 0.05). The mean HOMA-1IR and HOMA-2IR in patients with MetS were 4.90 ± 3.57 and 2.26 ± 1.42, respectively.

**Table 1:** Clinical and biochemical characteristics in control subjects and in patients with metabolic syndrome

Variables	Healthy Control (*n*= 47)	MetS (*n*= 47)	*P* value
**Age (years)**	43.72 ± 9.04	43.70 ± 11.75	-
**Sex, male/female**	36:11	36:11	-
**BMI (kg/m**^2^)	23.49 ± 3.15	28.70 ± 3.54	<0.001
**Waist circumference (cm)**	79.68 ± 10.08	96.52 ± 7.72	<0.001
**Hip circumference (cm)**	93.78 ± 5.21	101.51 ± 6.12	<0.001
**WHR**	0.85 ± 0.07	0.95 ± 0.05	<0.001
**Total betatrophin (ng/ml)**	1.31 ± 1.08	1.20 ± 0.79	0.524
**Full-length betatrophin (pg/ml)**	356.64 ± 287.92	694.84 ± 365.51	<0.001
**HbA1c (%)**	5.49 ± 0.34	6.54 ± 1.33	<0.001
**FPG (mmol/L)** ^&^	5.13 ± 1.10	6.31 ± 1.32	<0.001
**TG (mmol/L)** ^&^	1.35 ± 1.78	2.57 ± 1.91	<0.001
**TC (mmol/L)**	4.89 ± 0.77	5.48 ± 1.23	0.006
**HDL-C (mmol/L)**	1.49 ± 0.56	1.19 ± 0.35	0.003
**LDL-C (mmol/L)**	2.67 ± 0.52	2.90 ± 0.95	0.114
**Creatinine (umol/L)**	79.66 ± 13.79	79.87 ± 18.73	0.926
**eGFR (mL/min/1.73 m**^2^)	96.87 ± 11.61	97.82 ± 19.28	0.694
**UA (μmol/L)**	348.70 ± 92.38	440.55 ± 119.13	<0.001
**ALT (IU/L)**	23.80 ± 11.64	51.62 ± 38.40	<0.001
**AST (IU/L)**	23.39 ± 4.83	37.96 ± 21.42	<0.001
**ALP (IU/L)**	77.06 ± 20.58	79.47 ± 20.31	0.597
**ALB (g/L)**	47.71 ± 2.44	46.47 ± 3.22	0.012
**TBIL (umol/L)** ^&^	14.13 ± 1.45	13.18 ± 1.45	0.376
**DBIL (umol/L)**	4.78 ± 2.01	4.06 ± 1.60	0.054
**IBIL (umol/L)**	10.33 ± 4.61	10.07 ± 4.67	0.773
**GGT (IU/L)**^&^	22.91 ± 2.09	46.77 ± 2.19	<0.001

All values are given as mean ± SD.

^&^ indicates variables been log-transformed before paired-samples *t* test.

Abbreviations: IU, international unit.

### Circulating betatrophin levels

**Figure 2:**
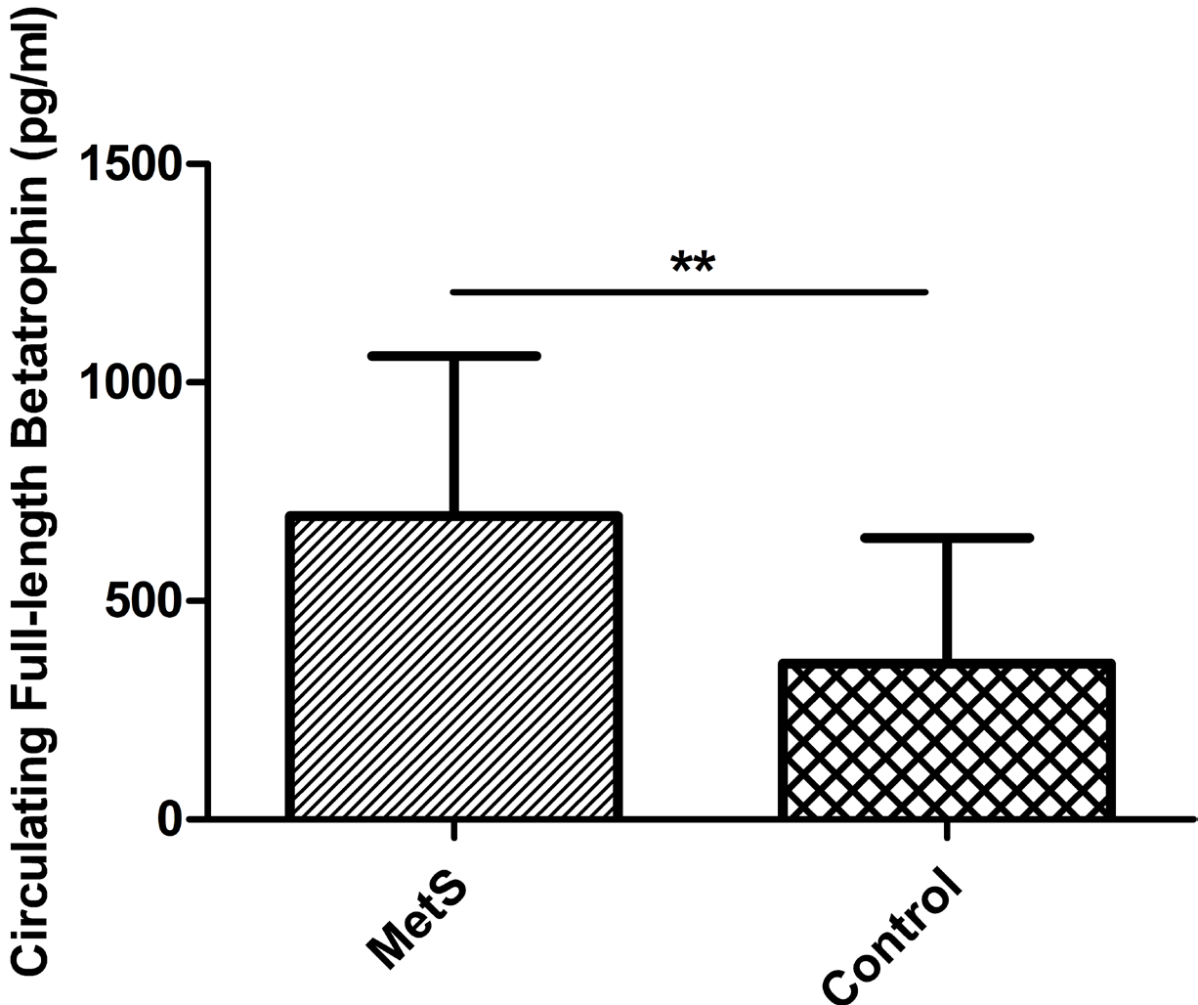
Circulating full-length betatrophin levels in patients with metabolic syndrome (*n* = 47) and healthy controls (*n* = 47) Error bar refers to standard deviation. ** < 0.001.

As shown in Figure 2, full-length betatrophin levels were higher in patients with MetS compared to those of controls with statistical significance (694.84 ± 365.51 pg/ml *versus* 356.64 ± 287.92 pg/ml; *P* < 0.001). While there was no significant difference of total betatrophin levels between the two groups (1.20 ± 0.79 ng/ml *versus* 1.31 ± 1.08 ng/ml; *P* = 0.524).

**Figure 3:**
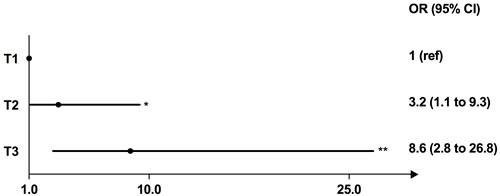
Association of betatrophin with metabolic syndrome Odds ratio (OR) for having metabolic syndrome according to the tertiles of the rank orders of circulating full-length betatrophin levels (reference: the lowest tertile). Abbreviations: T, tertile. * < 0.05; ** < 0.001.

As shown in Figure 3, binary logistic regression analysis of full-length betatrophin indicated subjects in the highest tertile and the intermediate tertile of betatrophin had significantly higher risks for developing MetS compared with those in the lowest tertile (highest *versus* lowest: OR, 8.6, 95% CI 2.8-26.8, *P* < 0.001; intermediate *versus* lowest: OR, 3.2, 95% CI 1.1-9.3, *P* < 0.05, respectively). The SPSS binary logistic regression output is shown in Supplementary Table 1.

### Correlations of betatrophin with clinical parameters

Among the patients with MetS, a significant correlation was found between full-length betatrophin and FPG (r = 0.357, *P* = 0.014) and 2-hour plasma glucose (2hPG) (r = 0.38, *P* = 0.008). Both correlations remain significant even after adjustment for age, sex and BMI (FPG, adjusted r = 0.350, *P* = 0.020; 2hPG, adjusted r = 0.372, *P* = 0.013, respectively) (Table 2). No significant correlation between full-length betatrophin and any other clinical parameters was detected (Table 2). Circulating total betatrophin was not correlated with these variables among the patients with MetS (Supplementary Table 2).

**Table 2:** Univariate and partial correlations with circulating full-length betatrophin levels in patients with metabolic syndrome

	Betatrophin *r*	*P* value	Betatrophin (age, sex and BMI adjusted) *r*	*P* value
Age	−0.039	0.797	-	-
BMI	−0.064	0.670	-	-
Waist circumference	0.003	0.981	0.049	0.750
Hip circumference	−0.127	0.396	−0.115	0.456
WHR	0.151	0.311	0.139	0.368
HbA1c	0.246	0.095	0.239	0.118
FPG^&^	0.357	0.014	0.350	0.020
TG^&^	0.204	0.168	0.177	0.251
TC	−0.089	0.553	−0.124	0.424
HDL-C	−0.19	0.200	−0.180	0.242
LDL-C	−0.200	0.178	−0.210	0.171
Creatinine	0.136	0.361	0.105	0.496
eGFR	−0.127	0.396	−0.171	0.268
UA	0.050	0.739	−0.002	0.988
ALT	−0.089	0.554	−0.117	0.450
AST	−0.114	0.447	−0.144	0.350
ALP	0.212	0.153	0.219	0.154
GGT^&^	0.233	0.115	0.234	0.126
ALB	−0.207	0.162	−0.267	0.079
TBIL^&^	0.053	0.607	0.089	0.397
DBIL	0.058	0.699	0.078	0.613
IBIL	0.163	0.275	0.167	0.280
HOMA1-IR	0.208	0.16	0.235	0.124
1/ HOMA1-IR	−0.141	0.343	−0.155	0.314
HOMA1-β (%)	−0.014	0.927	−0.006	0.969
HOMA2-%B	−0.095	0.523	−0.097	0.531
HOMA2-%S	−0.077	0.609	−0.086	0.578
HOMA2-IR	0.125	0.403	0.154	0.317
1/ HOMA2-IR	−0.078	0.605	−0.087	0.572
Glucose_120_	0.38	0.008	0.372	0.013
Insulin_0_^&^	0.070	0.640	0.090	0.563
Insulin_120_	−0.037	0.805	−0.051	0.741

^&^ indicates log-transformed variables.

Abbreviations: HOMA1-IR, homeostasis model assessment 1 to estimate insulin resistance; HOMA1-β, homeostasis model assessment 1 to estimate β-cell function; HOMA2-%B, homeostasis model assessment 2 to estimate β-cell function; HOMA2-IR, homeostasis model assessment 2 to estimate insulin resistance; HOMA2-%S, homeostasis model assessment 2 to estimate insulin sensitivity.

## DISCUSSION

In the current study, we detected the circulating betatrophin levels by using ELISA kits from two different manufacturers, and compared the data between MetS patients and age-, sex-matched controls. We have demonstrated that serum full-length but not total betatrophin levels were higher in patients with MetS than those in healthy controls.

Our data were supported by the work of Abu-Farha et al [30]. This unmatched nested case-control study with large sample size in West and South Asia indicated a 2.4 fold increased risk of MetS in subjects of the highest full-length betatrophin level tertile compared to those of the lowest tertile. To be noted, we used a group of strictly matched “super healthy” controls, when they used mixed controls with one or more metabolic disorders which did not meet the criteria of MetS. The difference of control selection may help explain their slighter association compared with our study.

Nevertheless, our findings were not consistent with another recent published study performed by Crujeiras et al [22] in which serum total betatrophin levels of obese-MetS subjects were higher than those of normal-weight subjects, while our study did not detect a significant difference between MetS patients and healthy controls. The disparity may be due to differences in anthropometric characters and medication usage. All MetS patients enrolled in Crujeiras’ study were obese Caucasian and some of the patients were receiving anti-diabetic medications. Our MetS were all drug-naïve Chinese Han population, 53% of which were not obese. Whether oral hypoglycemic agents will affect serum betatrophin concentration remains unclear. Based on our findings, we hypothesized that full-length and total betatrophin may exert different effects under various physiological and pathological conditions, which needs to be validated by further studies.

Our results also showed that fasting serum full-length betatrophin levels were positively correlated with FPG and 2hPG. Consistent with our finding, previous studies conducted by different groups also showed positive correlation between full-length betatrophin and FPG or 2hPG [17, 18]. The association between full-length betatrophin and glucose parameters confirmed the potential role of betatrophin in the development of T2DM, which was the dominant component of MetS. To be noted, circulating total betatrophin level was not found to be associated with any of these parameters in our study. It is different from Crujeiras et al.'s results [22]. Apparently, it was the cleavage of circulating betatrophin that contributed to the pathogenesis of these metabolic disorders. Further investigations are required to illustrate these mechanisms.

Our data did not find the association between circulating full-length betatrophin level with lipid profiles, transaminases and other parameters, which were suggested by previous reports [17, 31]. In a recent study, urine albumin / creatinine ratio (uACR) was considered to be associated with the circulating betatrophin level [32]. However, uACR was not measured in our study, and could be investigated in the future analysis.

Our study had several strengths. Firstly, both full-length and total betatrophin concentrations were measured in the same study population. Secondly, our study used age- and sex- matched controls making two groups more comparable. Thirdly, “super-healthy” criteria made a homogenous control group. Fourthly, all participants in our study were drug-naïve, and potential confounding factors were adjusted.

We also have some limitations. Firstly, this study cannot prove causality due to its cross-sectional study design. Secondly, despite the number of included participants reached the calculated sample size, it did not allow further subgroup analysis due to the limited power. Nevertheless, “super-healthy” matching controls and drug naïve patients had adjusted major confounding factors during the study design.

In conclusion, our study has demonstrated that circulating full-length betatrophin concentration but not total betatrophin concentration was higher in drug-naïve MetS patients and positively correlated with both fasting and postprandial blood glucose. Further studies are required to explore the potential role of circulating betatrophin and its cleavage in the pathogenesis of MetS.

## MATERIALS AND METHODS

### Study design and subjects

We recruited patients with MetS from the Outpatient Clinic by the Department of Endocrinology and Metabolism of West China Hospital and healthy volunteers as controls from the West China Hospital Physical Examination Center. All participants were enrolled between July 2015 and December 2015. General inclusion criteria for both groups were as follows: (1) aged between 18 and 70 years old; (2) informed consent to participate in the study; (3) adequate serum was collected for this study. A specific inclusion criterion for the MetS group was being diagnosed with MetS based on clinical and laboratory assessments according to the 1998 WHO consensus criteria [33]. Age and sex matched healthy subjects were recruited reaching the following criteria: normal body weight (BMI ranged from 18.5 to 23.9 kg/m^2^) without self-reported metabolic disorders of MetS, such as hypertension, dyslipidemia, diabetes mellitus, impaired glucose tolerance or impaired fasting glucose. Based on self-reported information, the participants with following conditions were excluded from both groups: (1) presence of acute infectious diseases, such as acute upper respiratory tract infection; (2) hepatic cirrhosis or ongoing dialysis; (3) congestive heart failure; (4) known malignancy; (5) psychological disorders; (6) pregnancy; (7) taking medications for dyslipidemia, hyperglycemia, hypertension, insulin resistance or obesity.

All subjects enrolled gave their informed consent to participate in this study. The protocol for this study was approved by the ethics committee at our hospital.

### Sample size calculation

The sample size was calculated for a 1:1 paired sample study with a power of 90% and a two-sided statistical significance level of 0.05. The standard deviation of circulating full-length betatrophin concentration was based on a previous publication of T2DM [26]. Setting a non-response rate of 20% resulted in a total sample size of 44 required for this study.

### Anthropometric measurements

All participants provided their medical history and received physical examination. Anthropometric and body composition measurements were performed within two days when blood samples were collected. Weight and height were measured when the participants were required to wear light clothes and barefooted after defecation. BMI was calculated as body weight in kilograms divided by the square of the height in meters. Waist circumference was measured at the narrowest point between the rib cage and the iliac crest. Hip circumference was measured at the widest point over the hipshot. WHR was calculated as waist circumference in centimeters divided by hip circumference in centimeters.

### Biochemical measurements

All blood samples were collected after overnight fasting for at least 8 hours. Oral glucose tolerance test (OGTT) with 75g glucose was performed in MetS patients. Insulin levels were measured by electrochemiluminescence immunoassay (ECLIA) method (Elecsys 2010, Roche Diagnostics, Mannheim, Germany); HbA1c was determined by a method based on high-performance liquid chromatography (HPLC) which was approved by National Glycohemoglobin Standardization Program (NGSP) (HLC-723 G8, Tosoh Corporation, Japan); Plasma glucose, TC, TG, LDL-C, HDL-C, ALT, AST, GGT, ALP, ALB, TBIL, DBIL, IBIL, creatinine, UA were measured on an automatic biochemistry analyzer (Modular P800, Roche Diagnostics GmbH, Germany) according to standard laboratory procedures. The homeostatic model assessment (HOMA) is used to quantify insulin resistance and beta-cell function. The HOMA1-IR index was calculated by the following formula:

HOMA1-IR = fasting plasma insulin (μU/mL) × fasting plasma glucose (mmol/L) / 22.5, $HOMA1-\beta \left(\%\right)=\frac{20\times fasting\;plasma\;insulin\left(\mu U/ml\right)}{fasting\;plasma\;glu\cos e\left(mmol/L\right)-3.5}\left(\%\right)$[34]. The HOMA2-IR index was calculated by the program HOMA Calculator v2.2.3 [35]. The estimated glomerular filtration rate (eGFR) was calculated by Modification of Diet in Renal Disease (MDRD) equation.

### Circulating betatrophin measurement

Circulating full-length betatrophin concentration was determined using a commercially available ELISA kit (catalog number E11644h; Wuhan Eiaab Science, Wuhan, China) with the intra- and inter-assay coefficient of variance (CV) ≤4.8% and ≤7.2% respectively. Total betatrophin level (including full-length and C-terminal fragment of betatrophin) was assessed by another ELISA kit (catalog number EK-051-55, Phoenix Pharmaceuticals, Phoenix, USA) [36]. The intra- and inter-assay CV were < 10% and < 15% respectively. All samples were analyzed in duplicates according to the manufacturer's instructions. Samples with CV > 15% were measured repeatedly.

### Statistical analysis

Distribution of the data was tested by the Kolmogorov-Smirnov test. FPG, TG, GGT, TBIL and insulin (0 min) values were logarithmically transformed before analysis due to their non-normal distribution. After transformation, all data were normally distributed and presented as mean ± standard deviation (SD). Comparisons of quantitative variables among groups were performed by paired-samples *t* test. Categorical variables were examined by χ^2^ test. Correlations between variables were analyzed by Pearson's correlation coefficients (*r*) among patients with MetS. Participants were stratified into tertiles according to the rank orders of their serum full-length betatrophin levels in the overall population. Odds ratios (ORs) with 95% confidence intervals (CIs) were estimated by performing binary logistic regression analysis to assess circulating betatrophin tertile on the risk of MetS. SPSS software version 20.0 (SPSS Inc., Chicago, IL) was used for all statistical analysis. A *P* value < 0.05 was considered statistically significant.

## SUPPLEMENTARY MATERIALS TABLES




