# Supplementary material for: Increased circulating full-length betatrophin levels in drug-naïve metabolic syndrome
Source: Oncotarget. 2017 Feb 4;8(11):17510–7. doi: 10.18632/oncotarget.15102 (PMC5392266; doi:10.18632/oncotarget.15102)
Supplement: Supplementary file 1 [file oncotarget-08-17510-s001.docx]

**Supplementary table 1: Result of binary logistic regression model**

|  | B | S.E. | Wald | df | Sig. | Exp(B) | 95% C.I. for EXP(B) | |
| --- | --- | --- | --- | --- | --- | --- | --- | --- |
|  |  |  |  |  |  |  | **Lower** | **Upper** |
| Step 1^a^ Betatrophin tertile |  |  | 13.892 | 2 | 0.001 |  |  |  |
| Betatrophin tertile (1) | 1.163 | 0.544 | 4.573 | 1 | 0.032 | 3.200 | 1.102 | 9.292 |
| Betatrophin tertile (2) | 2.155 | 0.579 | 13.852 | 1 | 0.000 | 8.625 | 2.773 | 26.825 |
| Constant | -1.099 | 0.408 | 7.242 | 1 | 0.007 | 0.333 |  |  |

1. Variable(s) entered on step1: betatrophin tertile.
