# Supplementary material for: Increased circulating full-length betatrophin levels in drug-naïve metabolic syndrome
Source: Oncotarget. 2017 Feb 4;8(11):17510–7. doi: 10.18632/oncotarget.15102 (PMC5392266; doi:10.18632/oncotarget.15102)
Supplement: Supplementary file 2 [file oncotarget-08-17510-s002.docx]

**Supplementary table 2: Univariate and partial correlations with circulating total betatrophin levels in patients with metabolic syndrome**

|  | Betatrophin  r | *P* value | Betatrophin (age, sex and BMI adjusted)  r | *P* value |
| --- | --- | --- | --- | --- |
| Age | 0.054 | 0.719 | - | - |
| BMI | 0.031 | 0.838 | - | - |
| Waist circumference | 0.068 | 0.651 | 0.167 | 0.277 |
| Hip circumference | 0.044 | 0.769 | -0.005 | 0.972 |
| WHR | 0.046 | 0.759 | 0.171 | 0.268 |
| HbA1c | 0.009 | 0.953 | 0.037 | 0.814 |
| FPG^&^ | -0.083 | 0.579 | -0.044 | 0.777 |
| TG^&^ | 0.169 | 0.256 | 0.241 | 0.114 |
| TC | 0.074 | 0.623 | 0.119 | 0.443 |
| HDL-C | -0.087 | 0.561 | -0.116 | 0.454 |
| LDL-C | -0.168 | 0.260 | -0.163 | 0.291 |
| Creatinine | 0.05 | 0.737 | 0.163 | 0.289 |
| eGFR | -0.072 | 0.629 | -0.101 | 0.514 |
| UA | -0.099 | 0.507 | -0.030 | 0.846 |
| ALT | -0.009 | 0.950 | -0.002 | 0.988 |
| AST | 0.009 | 0.953 | 0.035 | 0.822 |
| ALP | 0.007 | 0.960 | -0.005 | 0.974 |
| GGT^&^ | -0.143 | 0.337 | -0.098 | 0.525 |
| ALB | 0.096 | 0.521 | 0.146 | 0.343 |
| TBIL^&^ | -0.050 | 0.631 | -0.033 | 0.752 |
| DBIL | -0.248 | 0.093 | -0.274 | 0.072 |
| IBIL | -0.140 | 0.349 | -0.126 | 0.415 |
| HOMA1-IR | 0.054 | 0.721 | 0.104 | 0.502 |
| 1/ HOMA1-IR | -0.053 | 0.723 | -0.094 | 0.544 |
| HOMA1-β (%) | -0.027 | 0.859 | -0.024 | 0.878 |
| HOMA2%B | 0.033 | 0.825 | 0.032 | 0.836 |
| HOMA2%S | -0.084 | 0.574 | -0.127 | 0.412 |
| HOMA2-IR | 0.071 | 0.635 | 0.119 | 0.441 |
| 1/ HOMA2-IR | -0.084 | 0.576 | -0.126 | 0.414 |
| Glucose_120_ | -0.128 | 0.392 | -0.087 | 0.573 |
| Insulin_0_^&^ | -0.068 | 0.650 | 0.116 | 0.454 |
| Insulin_120_ | 0.023 | 0.876 | 0.047 | 0.761 |

^&^ indicates log-transformed variables.

Abbreviations: HOMA1-IR, homeostasis model assessment 1 to estimate insulin resistance; HOMA1-β, homeostasis model assessment 1 to estimate β-cell function; HOMA2-%B, homeostasis model assessment 2 to estimate β-cell function; HOMA2-IR, homeostasis model assessment 2 to estimate insulin resistance; HOMA2-%S, homeostasis model assessment 2 to estimate insulin sensitivity.
